# Blood proteins self-assembly, staphylococcal enterotoxins-interaction, antibacterial synergistic activities of biogenic carbon/FeSO_4_/Cu/CuO nanocomposites modified with three antibiotics

**DOI:** 10.1186/s13065-024-01115-4

**Published:** 2024-01-23

**Authors:** Mehran Alavi, Nasser Karimi

**Affiliations:** 1 Nanobiotechnology Department, Faculty of Innovative Science and Technology, Razi University, Kermanshah, Iran; 2 Department of Biology, Faculty of Science, Razi University, Kermanshah, Iran

**Keywords:** Albumin, Hemoglobin, Self-assembly, Antibacterial synergism, carbon/FeSO_4_/Cu/CuO nanocomposites, Antibiotic-functionalized nanocomposites

## Abstract

**Introduction:**

Nanocomposites based on copper, iron, and carbon materials are novel nanomaterials with both antibacterial and biocompatibility properties considerable to fight against multidrug-resistant bacteria.

**Methods:**

In this study, phytogenic carbon/FeSO_4_/Cu/CuO nanocomposites modified by three antibiotics including tetracycline, amoxicillin, and penicillin were employed to hinder antibiotic resistant bacteria of *Escherichia coli*, *Staphylococcus aureus*, and *Pseudomonas aeruginosa*. Interaction of albumin and hemoglobin as major blood proteins with these nanocomposites were evaluated by SEM, FTIR, and AFM techniques. As in silico study, molecular docking properties of staphylococcal enterotoxin toxin A and B with (Z)-α-Bisabolene epoxide, (E)-Nerolidol, α-Cyperone, daphnauranol C, nootkatin, and nootkatone as major secondary metabolites of Daphne mucronata were obtained by AutoDock Vina program.

**Results:**

Physicochemical characterization of nanocomposites showed (Zeta potential (− 5.09 mV), Z-average (460.2 d.nm), polydispersity index (0.293), and size range of 44.58 ± 6.78 nm). Results of both in vitro and in silico surveys disclosed significant antibacterial activity of antibiotic functionalized carbon/FeSO_4_/Cu/CuO nanocomposites compared to antibiotics alone towards Gram-negative and Gram-positive bacteria.

**Conclusion:**

Synergistic activity of bio-fabricated carbon/FeSO_4_/Cu/CuO nanocomposites with antibiotics may be affected by main parameters of concentration and ratio of antibacterial agents, physicochemical properties of nanocomposites, bacterial type (Gram-negative or Gram-positive), antibacterial mechanisms, and chemical structure of antibiotics.

## Introduction

Multidrug-resistant strains in bacteria are augmenting due to the misuses of the various antibiotics ranges, failing to overcome bacteria at the planktonic or biofilm stages threatening human health [1–4]. For instance, methicillin-resistant *S. aureus* (MRSA) a main agent of hospital-acquired infections has the resistance ability to the antibiotics of methicillin, macrolides, tetracycline, chloramphenicol, lincosamides, and aminoglycosides [5]. Nanotechnology has presented novel strategies such as functionalized nanomaterials with the considerable efficiency encompassing a large surface area and higher reactivity relative to bulk materials [6, 7]. As a common way of nanomaterial classification, organic, inorganic, and organic/inorganic complex can be prepared by chemical, physical, and biological approaches [8–10]. Plants, bacteria, fungi, alga, lichens, and viruses may be employed to biosynthesize eco-friendly nanomaterials, wherein metal, metal oxide nanoparticles (NPs) or nanocomposites (NCs) as inorganic nanomaterials have obtained more attention to overcome pathogens and cancer cells [11]. Antibacterial mechanisms of these nanomaterials are resulted from releasing of metal ions followed by generation of reactive oxygen species (ROS) in bacteria medium. Cellular envelops of bacteria are sensitive to these ions and ROS, which can be interrupted followed by biological macromolecules depletion. Metal ions and ROS can interact with DNA and proteins specifically enzymes lead to damage or inefficiency of these macromolecules [12]. In addition, various antibiotics may be exploited to functionalize and synergize antibacterial activity of NPs or NCs against resistant bacteria specifically multidrug-resistant bacteria [13]. It is worth noting that in comparison with AgNPs, which have significant antibacterial activity, Cu or CuO NPs also have showed antibacterial effects with advantages of appropriate biocompatibility. Additionally, these NPs as p-type semiconductor materials have bandgap of 1.2 eV, which is suitable to harvesting light particularly in catalytic, electrical, and optical tools [14]. Therefore, these types of metallic NPs can be considered as safe antibacterial agents in various formulations [15].

There are various chemical, physical or biological methods to prepare NCs containing copper or copper oxide NPs having antimicrobial activity [16]. Biological synthesis of metal/metal oxide NPs can be carried out by various extracts of plants, bacteria, fungi, and lichens with desirable biocompatibility property compared to physical or chemical methods [17, 18]. In the case of plant extracts, several parts including fruits, leaves, stems, roots, and seeds having primary and secondary metabolites can participate in the preparation of metal/metal oxide NPs [19–21]. In compression to chemical and physical methods, secondary metabolites of plants can synergize therapeutic activities, specifically antibacterial activity with lower cytotoxicity [22–24]. As a main advantage, by coating these biocompatible NPs with antibiotics such as penicillin, ampicillin, and tetracycline, synergistic effects can be resulted against multidrug-resistant (MDR) bacteria [4].

Copper (Cu) or copper oxide (CuO) NCs composed of carbon, sulfur, and iron elements have shown significant antibacterial against Gram-negative and Gram-positive bacteria [25–27]. In green synthesis, in addition to antibacterial ability, biocompatibility of these NPs can be increased in safe level suitable for human physiological conditions [28–30]. In this regard, for measuring of self-assembly property, interaction of two main proteins of blood including albumin and hemoglobin with NPs or NCs is valuable [31]. Therefore, in the present study, antibacterial activity and hemoglobin/albumin interaction of antibiotic (penicillin, tetracycline, and amoxicillin) functionalized phytogenic carbon/FeSO_4_/Cu/CuO NCs (CuNCs) prepared by leaves/flowers aqueous extract of *Daphne mucronata* (medicinal plant species with antimicrobial and anticancer activities [32]) were evaluated by in vitro and in silico studies. For in silico part, docking properties of staphylococcal enterotoxin toxin A and B with (*Z*)-α-Bisabolene epoxide, (*E*)-Nerolidol, α-Cyperone, daphnauranol C, nootkatin, and nootkatone as major secondary metabolites of *D. mucronata* were surveyed by AutoDock Vina program.

## Materials and methods

### Materials

Copper (II) sulfate pentahydrate (CuSO_4_.5H_2_O), Whatman No. 40 filter paper, penicillin G sodium (C_16_H_17_N_2_NaO_4_S), amoxicillin (C_16_H_19_N_3_O_5_S), tetracycline hydrochloride (C_22_H_24_N_2_O_8_·HCl), albumin, and hemoglobin proteins were prepared from Sigma-Aldrich Company.

### Methods

#### Preparation of leaves/flower aqueous extract

Samples of leaves and flowers of *D. mucronata* were collected from the Amrooleh mountainous area, grid reference 36°43′04′N 47°08′02′E, 40 km north of Sahneh town in Kermanshah province, west of Iran during July 2019 and identified by Hosein Maroofi in the central herbarium of Kurdistan agriculture and resource research center (Sanandaj, Kurdistan) by comparing them with the herbarium species and by using the flora of Iranica. The voucher number (BD-55-03) was deposited for this species. After collecting and identification of plant species according to previous study, leaves/flowers aqueous extract of *D. mucronata* was obtained from 20 g of fresh leaves/flowers. The leaves/flowers surface was cleaned completely by running tap water and dried on a paper towel for 2 weeks at 25 °C temperature. For preparing fine powder, dried leaves/flowers were ground in a tissue grinder followed by mixing with 200 mL of double distilled water and boiling at 60–80 °C for 30 min. Whatman No. 40 filter paper was employed to purify resulted suspensions and due to further application, after collection of the filtered suspension, samples were stored at refrigerator at 4 °C [33].

#### Biogenic NCs and antibiotic-functionalized CuNCs preparation

A centrifugation at 5000 rpm for 30 min was applied to obtain supernatant of the aqueous leaves/flowers extract. For synthesis of phytogenic carbon/FeSO_4_/Cu/CuO NCs (CuNCs), the aqueous solution containing 100 mL of 0.1 M concentration of CuSO_4_.5H_2_O was stirred for 60 min. The prepared samples was mixed with plant aqueous extract in 1:9 ratio 25 °C under stirred condition for 30 min. For further purification of NCs, samples were centrifugated at 5000 rpm for 30 min. In order to measure spectra of NCs by UV–Vis spectroscopy, the supernatant having NCs was transferred to refrigerator and stored at 4 °C temperature in the dark condition. Reduction and stabilization of Cu^2+^ ions and CuNCs by leaf/flower aqueous extract of *D. mucronata* resulted in color change from blue to green colors. In addition to UV–Vis spectroscopy, physicochemical properties of phytogenic CuNCs including crystalline structure, size/shape and functional groups were indicated by X-ray powder diffraction (XRD), transmission electron microscopy (TEM) and Fourier-transform infrared (FTIR) spectroscopy. For synthesis of antibiotic-CuNCs complex, under stirring condition for 1 h at room temperature, tetracycline (30 µg/mL), amoxicillin (60 µg/mL), and penicillin (20 µg/mL) were separately mixed by 1 mg concentration of CuNCs. After centrifugation of mixture at 5000 ppm for 30 min and drying precipitation at 60 °C for 3 h, FTIR spectroscopy and scanning electron microscopy (SEM) technique were used to indicate formation of the samples [34, 35].

#### Antibacterial activities

##### Disc diffusion, MIC, MBC, and morphology of bacteria

*S. aureus* ATCC 43300, *P. aeruginosa* ATCC 27853, and *E. coli* ATCC 25922 as multidrug-resistant and sensitive bacterial strains were treated upon effect of leaf/flower aqueous extract, CuNCs, and CuNCs/antibiotic. According to previous study, Kirby-Bauer test method was used to determine the inhibition zone diameters (IZDs) around each disks [35]. MIC and MBC assays were carried out at amounts of 1000, 500, 250, 100, 50, 25 μg/mL of CuNCs, CuNCs/penicillin (CuNCs/Pe), CuNCs/amoxicillin (CuNCs/Am), and CuNCs/tetracycline (CuNCs/Te) for comparison of their minimum bacteriostatic and bactericidal concentrations [36]. Based on our previous study [37], the morphology of bacteria was determined under exposure of MIC concentration of CuNCs/Te. In this way, one drop from stationary phase of growth culture related to *E. coli* and *S. aureus* as sensitive bacteria was put on glass slides and after fixation and dehydration was observed via SEM technique.

#### Self-assembly of albumin/hemoglobin interacted with AgNPs/Te

Self-assembly properties of CuNCs/Te interacted with hemoglobin and albumin was determined by FTIR and SEM techniques. Human serum albumin and hemoglobin at 0.001 M were mixed by CuNCs/Te in Milli-Q water at 25 °C temperature under stirrer condition for half hour. Assembled albumin and hemoglobin around CuNCs/Te were casted separately on the silicon slides and dried at room temperature for 48 h. At the final step, three techniques involving FTIR spectroscopy, atomic force microscopy (AFM), and SEM techniques were exploited to indicate type of functional groups responded to CuNCs/Te/blood proteins complexes and their size/shape, respectively [38–40].

#### Molecular docking study

**Fig. 1:**
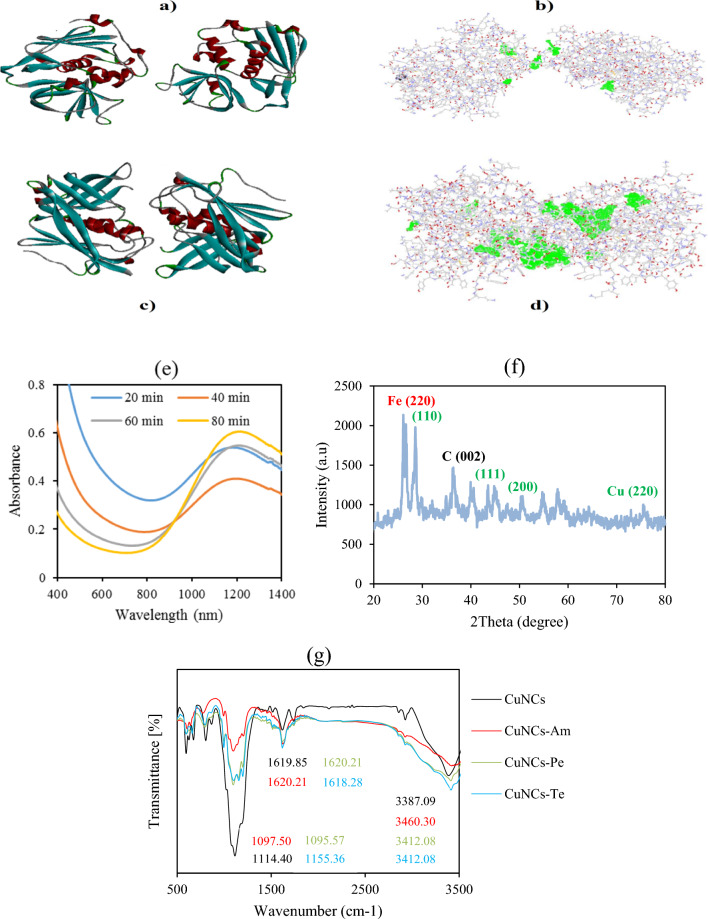
3D structures of staphylococcal enterotoxin A (ID: 1ESF) with related seven cavities (**a**, **b**) and enterotoxin B (ID: 1GOZ) by related ten cavities (**c**, **d**). Spectra for UV–Vis at the periodicity of 20 min for CuNCs (**e**), XRD with related indices of CuNCs (**f**), and FTIR for CuNCs, CuNCs-Am, CuNCs-Pe, and CuNCs-Te (**g**)

**Fig. 2:**
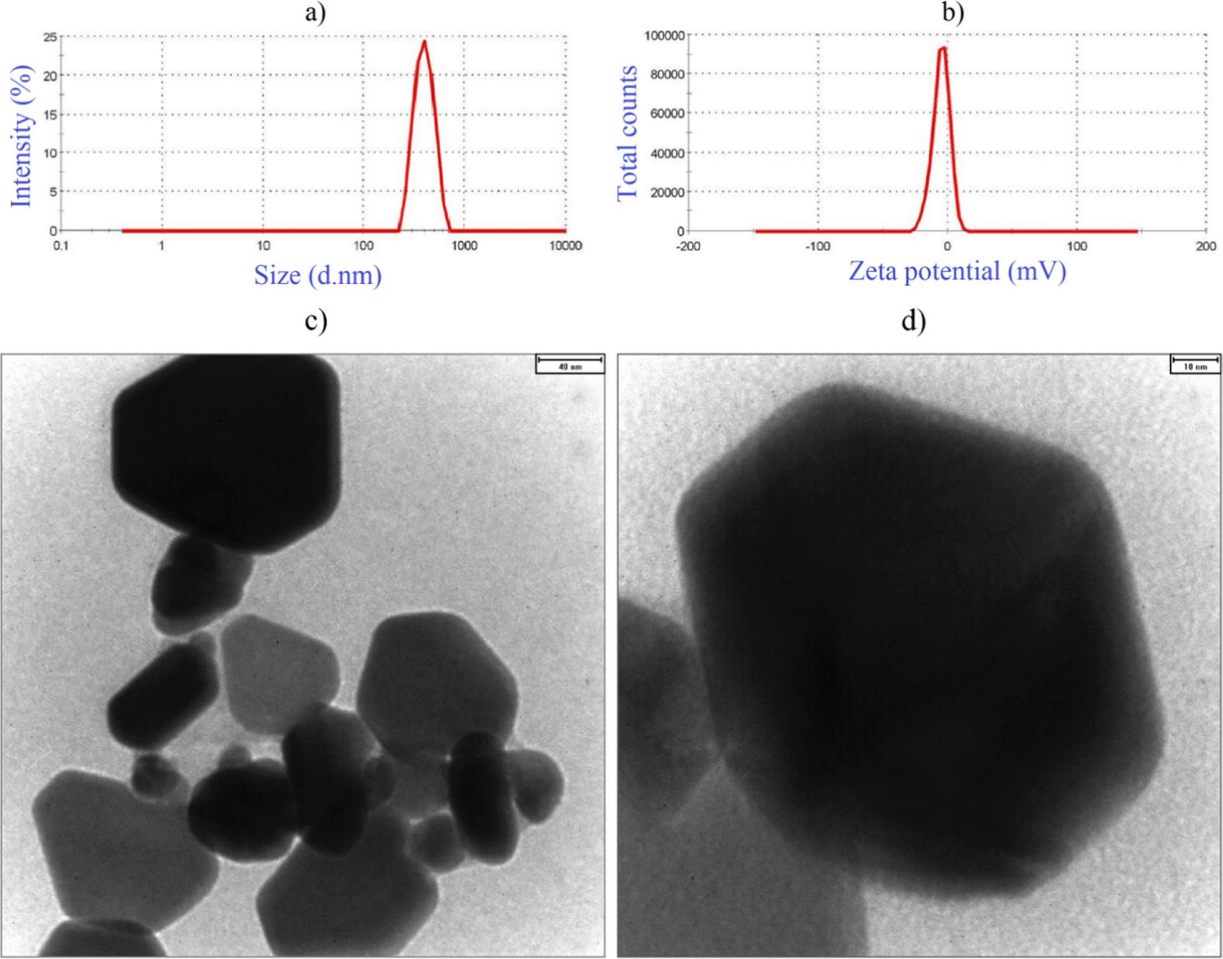
Size distribution (**a**), zeta potential distribution (**b**), and bright field TEM photographs in the scale bars of 40 nm (**c**) and 10 nm (**d**) for carbon/FeSO_4_/Cu/CuO NCs green synthesized by *D. mucronata*

PubChem and J-GLOBAL databases were used to download the SDF files of major secondary metabolites responded to essential oils of aerial parts of *D. mucronata* (Table 1) followed by converting these files to PDB file by Discovery Studio 2016. Optimization of resulted files was done by UCSF chimera 1.12 (Fig. 1a–f). 3D structures of two main virulence factors of *S. aureus* including staphylococcal enterotoxin A (ID: 1ESF) and enterotoxin B (ID: 1GOZ) were got from Protein Databank (PDB; http://www.rcsb.org) (Fig. 2a–d). AutoDock Vina program (version: 1.5.7) was applied to evaluate molecular docking properties between each staphylococcal enterotoxin A and enterotoxin A with (Z)-α-Bisabolene epoxide, (E)-Nerolidol, Nootkatone, α-Cyperone, Daphnauranol C, and Nootkatin, separately. Docking score and the root-mean-square deviation (RMSD) of each secondary metabolite with enterotoxin A and enterotoxin B was obtained via the HDOCK server (http://hdock.phys.hust.edu.cn/) [41].

**Table 1:** Oxygenated sesquiterpenes found as major secondary metabolites in essential oils of aerial parts of *D. mucronata* (https://pubchem.ncbi.nlm.nih.gov/)

Compound names	Molecular weight (g/mol)		Solubility
Nootkatone	218.33		Slightly soluble in water, 36 mg/L at 25 °C and soluble in alcohol and oil
Nootkatin	232.32		Insoluble or slightly soluble in water and soluble in chloroform
Daphnauranol C	232.323		Soluble in oils
α-Cyperone	218.33		Insoluble in water and slightly soluble in Dimethyl sulfoxide (DMSO)
(*E*)-Nerolidol	222.37		Insoluble in glycerol, slightly soluble in water, soluble in ethanol, in most oils and propylene glycol
(Z)-α-Bisabolene epoxide	220.35		Soluble in alcohol and slightly soluble in water

## Results and discussion

Obvious peak at the wavelength range of 1000–1200 nm was found for CuNCs at the periodicity of 20 min for 80 min, which showed the growth of NCs during this time (Fig. 1e). XRD spectrum revealed the availability of carbon, iron sulfate hydrate (FeSO_4_·xH_2_O), and tenorite (CuO) with copper (Fig. 1f). There were several functional groups indicated by FTIR for CuNCs, CuNCs-Am, CuNCs-Pe, and CuNCs-Te (Fig. 1g). C=C stretch and the C–H stretch were indicated for the bonds related to the phytochemicals such as carbohydrates, proteins, phenolic compounds, and alkaloids in the leaf extract of *Adhatoda vasica* Nees for green synthesis of CuO/carbon NCs [42]. The FT-IR spectra of CuFeS_2_ nanocrystals illustrated peaks at 2346 cm^−1^ related to stretching of C–S bonded to nanocrystal, significant peaks at 2918 cm^−1^ for stretching vibrations of C–H assigned responded to the capping ligand of 1-dodecathiol as well as 1446 and 1554 cm^−1^ for stretching vibrations of –CH_2_ and C–C, respectively [43]. Hydroxyl group was a common bond for creating the nano-complex of amoxicillin and ampicillin with AgNPs. Furthermore, formulation of ciprofloxacin-ZnONPs was based on interaction of carboxylic acid and aryl amine of antibiotic with ZnONPs [44]. As the main mechanism, reduction of metal ions such as Cu^2+^ and stabilization of NPs by secondary metabolites contribute to the synthesis of metal NPs and NCs [45, 46]. In the case of *D. mucronata*, nootkatin, daphnauranol C, and (E)-Nerolidol metabolites with having a hydroxyl group (Table 1) can contribute to the preparation of CuNCs.

### TEM micrographs and DLS analysis

As presented in Fig. 2a, b, Carbon/FeSO_4_/Cu/CuO NCs had zeta potential, Z-average, and PDI of − 5.09 mV, 460.2 d.nm, and 0.293 (mono dispersed particles), respectively. TEM images illustrated 44.58 ± 6.78 nm with various morphologies including triangular, rod, and hexagonal shapes (Fig. 2c, d). According to zeta potential result, aggregation stability is expected for these negative charged colloidal NCs. In a green synthesis way, bimetallic ZnO–CuO NCs fabricated by aqueous leaves extract of *Calotropis gigantean* plant species with three weight percentages of 75 wt%, 50 wt%, and 25 wt% of metallic salt (zinc nitrate hexahydrate) showed porous agglomerated morphology, irregular rod-shaped NPs, and honeycomb shape at agglomerated morphology, respectively [47]. Mixture of 0.01 M copper sulphate (CuSO_4_ 0.5H_2_O) solution and 1 g of the aqueous leaves extract of *A. vasica* was used to synthesize nanoflakes of CuO/carbon having a mean diameter of 7–11 nm [42]. The presence of natural compounds such as polyphenols, flavonoids, tannins, alkaloids, saponins, and reducing carbohydrates may be responsible for the reduction and stabilization of metal or metal oxides NCs, as it was confirmed for green synthesized CuO nanosheets (a mean size of 20 nm) by aqueous arial parts of *Rhazya stricta* plant species [48].

### Antibacterial activity

**Fig. 3:**
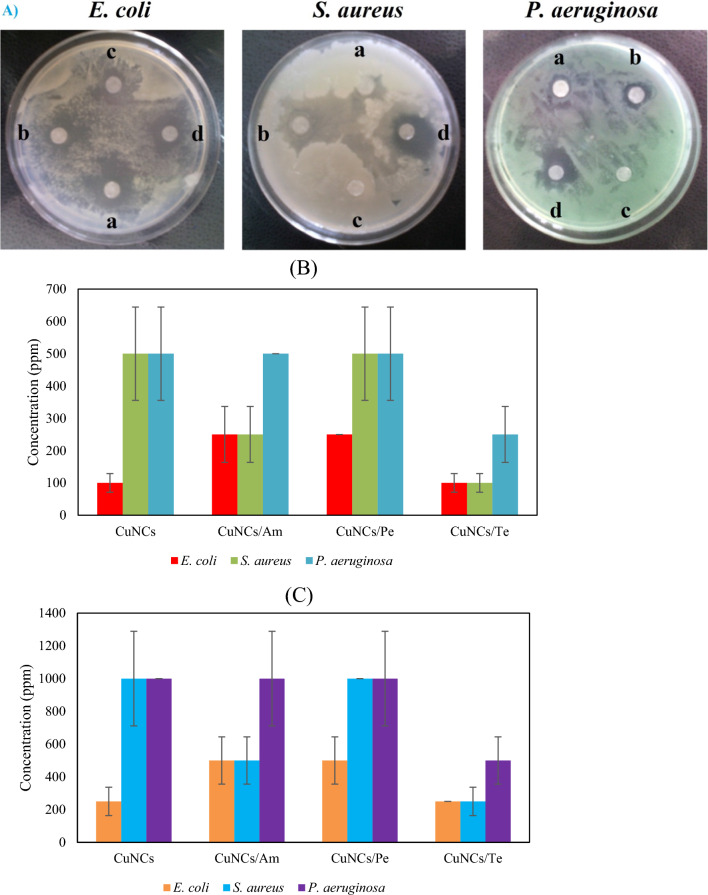

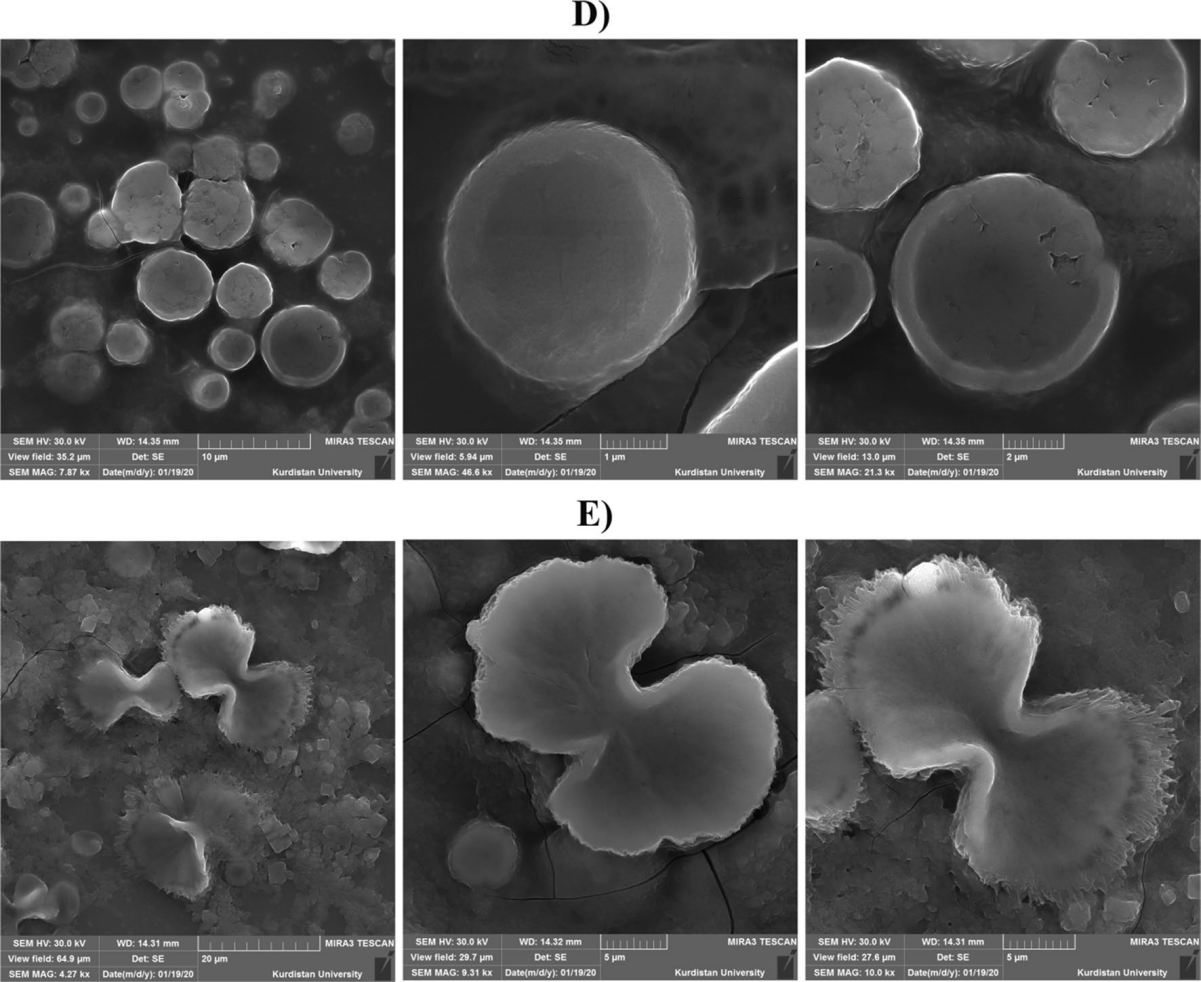
Different IZDs for CuNPs (**a**), CuNPs/Am (**b**), CuNPs/Pe (**c**), and CuNPs/Te (**d**) toward *E. coli*, *S. aureus*, and *P. aeruginosa*. **B** MIC and **C** MBC present results of NCs. SEM images illustrate control group (**D**) and cell wall deformation and disruption of *S. aureus* under CuNCs/Te treatment (**E**)

Higher antibacterial activity was found for CuNCs/Te toward *E. coli*, *P. aeruginosa*, and *S. aureus* as IZDs of 25.31, 23.07, and 27.21 mm, respectively (Table 2). *E. coli* as more sensitive bacteria revealed 27.37, 23.35, and 23.23 mm of IZD values upon stress of CuNCs, CuNCs/Am, CuNCs/Pe, sequentially statistically significant as *P* ≤ 0.05. Resistance of Gram-negative bacteria of *P. aeruginosa* towards CuNCs and CuNCs/Am was indicated as IZDs of 11.77 and 14.24 mm, wherein Gram-positive bacteria, *S. aureus,* had the lowest sensitivity to CuNCs/Pe as value of 1.03 mm. MIC and MBC assays confirmed disc diffusion test as lower bactericidal effects by values of 100, 100, and 250 ppm toward *E. coli*, *S. aureus*, and *P. aeruginosa*, respectively (Fig. 3B, C). Biogenic CuO/carbon NCs fabricated by the aqueous leaves extract of *A. vasica* showed zones of inhibition 11, 12, 14, and 11 mm against *P. aeruginosa*, *Klebsiella pneumoniae*, S*. aureus*, respectively [42]. Compared to bacterial sources for synthesis Cu or CuO NPs, *Streptomyces capillispiralis* Ca-1 strain isolated from *Convolvulus arvensis* as the endophytic actinomycete (Gram-positive mycelial bacteria) was exploited to prepare CuNPs with spherical shape and diameter of 3.6–59 nm and antifungal effect against *Pythium* spp., *Aspergillus niger*, and *Alternaria* spp., by the inhibition of 58.05%, 63.81, and 57.14, respectively at amount 20 mM of NPs [49]. In compared to ciprofloxacin antibiotic with zone of inhibition values of 21.3 and 21.3 mm, biosynthesized CuO NPs by *Actinomycete* sp. VITBN4 exhibited 14.3 and 15.6 mm against *S. aureus* and *Bacillus cereus*, respectively at 10 µg/mL concentration more than cell free supernatant of actinomycetes. The mean size, zeta potential, and shape of these NPs were 61.7 nm, − 31.1 mV, and spherical with aggregated form, respectively [50]. Optimized synthesis of CuNPs with face cubic center (fcc) crystal structure and average size of 56–73 nm was prepared by supernatant of culture medium of *Halomonas elongate* IBRC-M 10214, wherein IZDs for this NP were 10 and 8.7 mm toward *E. coli* and *S. aureus* [51].

**Table 2:** IZDs results of *E. coli*, *S. aureus* and *P. aeruginosa* under effect of CuNPs, CuNPs/Am, CuNPs/Pe, and CuNPs/Te

Bacteria	Antibacterial agents with IZD (mm) ± SD			
	CuNCs	CuNCs/Am	CuNCs/Pe	CuNCs/Te
*E. coli*	27.37 ± 0.85*	23.35 ± 0.70*	23.23 ± 0.92*	25.31 ± 1.14
*P. aeruginosa*	11.77 ± 1.53	14.24 ± 1.25	9.52 ± 0.51	23.07 ± 1.36
*S. aureus*	14.18 ± 1.20	26.92 ± 0.48	1.03 ± 1.05	27.21 ± 1.05

^*^Mark shows significant difference between highest and lowest IZD values for each NC at a level of *P* ≤ 0.05)

**Fig. 4:**
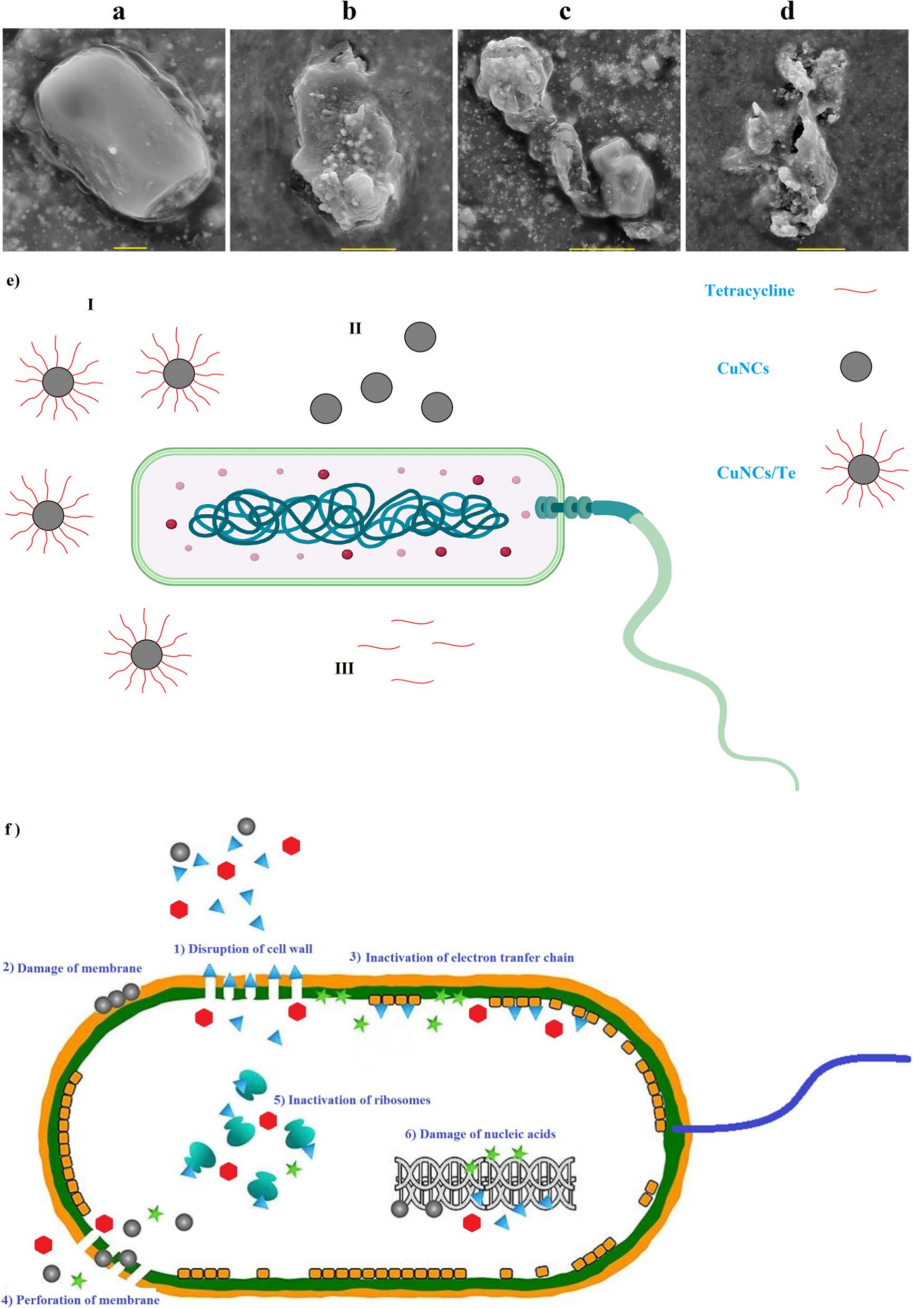
SEM photographs illustrate control group (**a**; scale bar of 2 µm) and cell envelop deformation and disruption of *E. coli* under CuNCs/Te treatment (**b**; scale bar of 5 µm, **c**, **d**; scale bar of 10 µm). **e** Schematic image showing three main probable pathways for antibacterial activity of CuNCs/Te (I), CuNCs (II), and tetracycline (III) **f** antibacterial mechanisms of CuNCs/Te and CuNCs [61]

NorA protein as NorA multidrug efflux pump in *S. aureus* was inhibited by interference of ciprofloxacin modified ZnONPs in function of this protein [44]. The interruptions of bacterial envelopes by NCs and break-down of bacterial enzymes are main antibacterial mechanisms for metallic NPs or NCs [11]. Surprisingly, at the genome level, both CuONPs and CuO in micro size can impact on the plasmid pUC19 as converting its supercoiled form to open circular one. However, in contrast to micro CuO, there was not any supercoiled form of plasmid on gel electrophoresis for CuONPs [52]. Figures 3 and 4 illustrate breaking of envelopes of *S. aureus* (thick peptidoglycan and cell membrane) and *E. coli* (outer membrane, thin peptidoglycan, and inner membrane) upon effect of CuNCs/Te, respectively. Synergistic effect was observed for the combination of 30 µg/mL of AgNPs biosynthesized via cyanobacteria of *Phormidium* sp. with chloramephnicol (0.5%) against methicillin-resistant *S. aureus* (MRSA) as IZDs of 28 mm compared to 20 and 15 mm for AgNPs and chloramphenicol, respectively [53]. In a similar investigation, AgNPs by a mean size of 29.8 nm were combined with several antibiotics including tetracycline, ampicillin, kanamycin, enoxacin, neomycin, and penicillin antibiotics. At the concentrations of 16, 8, 2, and 0.5 μM, significant bacterial inactivation of *Salmonella typhimurium* DT104 was observed as value of ~ 100% for AgNPs-tetracycline nanocompsites other AgNO_3_, AgNPs, and nanocomposites [54]. It should be noted that the therapeutic application of metal/metal oxide NPs can be affected by size distribution, shape, surface charge, surface chemistry, and capping agents [55–60]. As presented in Fig. 4e, three main pathways including antibacterial effects of CuNCs/Te and each CuNCs and tetracycline alone on bacteria may be considered, which most probable case can be antibacterial activity of CuNCs/Te.

### Molecular docking results

**Fig. 5:**
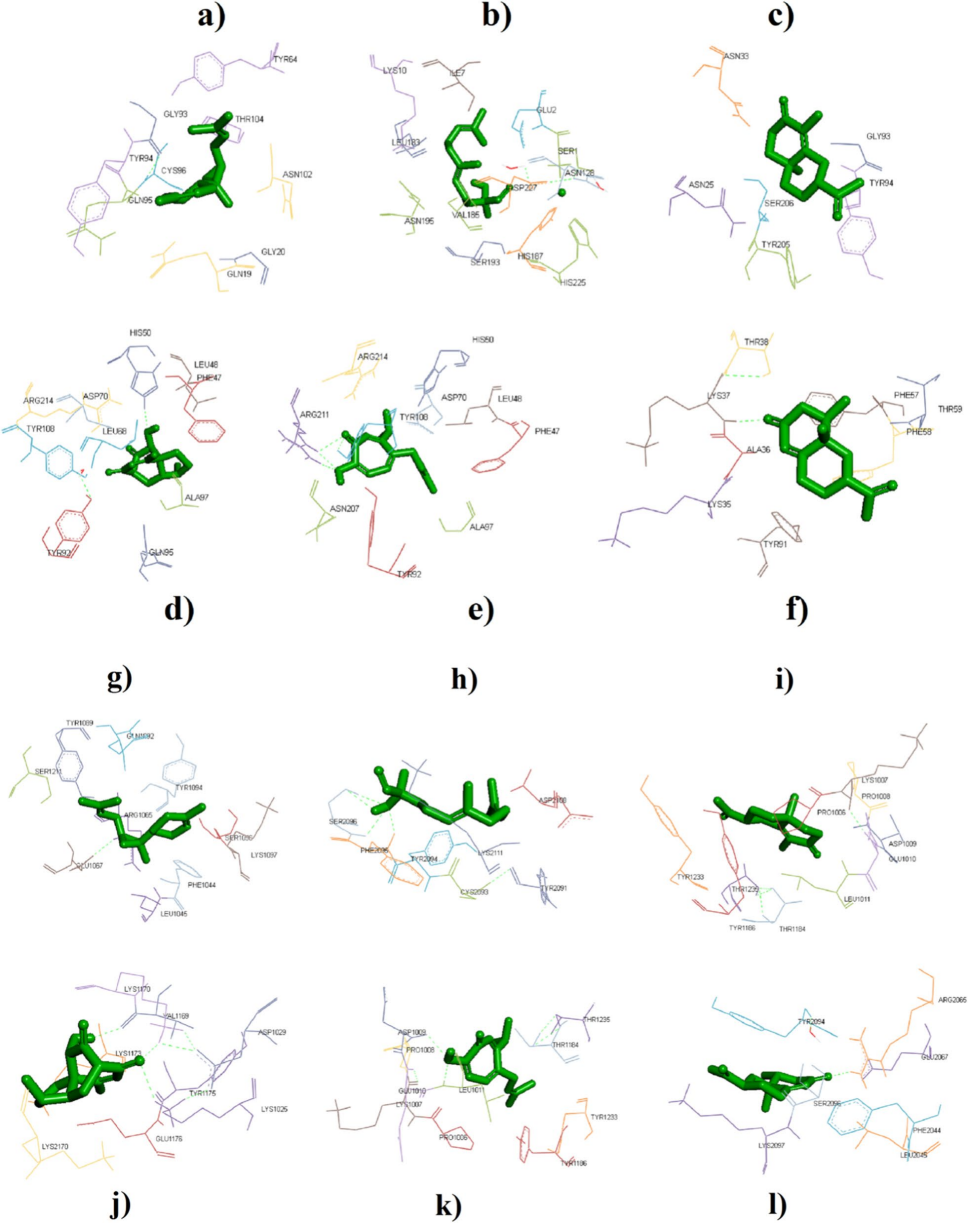
Interaction of (*Z*)-α-Bisabolene epoxide (**a**), (*E*)-Nerolidol (**b**), α-Cyperone (**c**), daphnauranol C (**d**), nootkatin (**e**), and nootkatone (**f**) with amino acids of enterotoxin A. Interaction of (*Z*)-α-Bisabolene epoxide (**g**), (*E*)-Nerolidol (**h**), α-Cyperone (**i**), daphnauranol C (**j**), nootkatin (**k**), and nootkatone (**l**) with amino acids of enterotoxin B

**Fig. 6:**
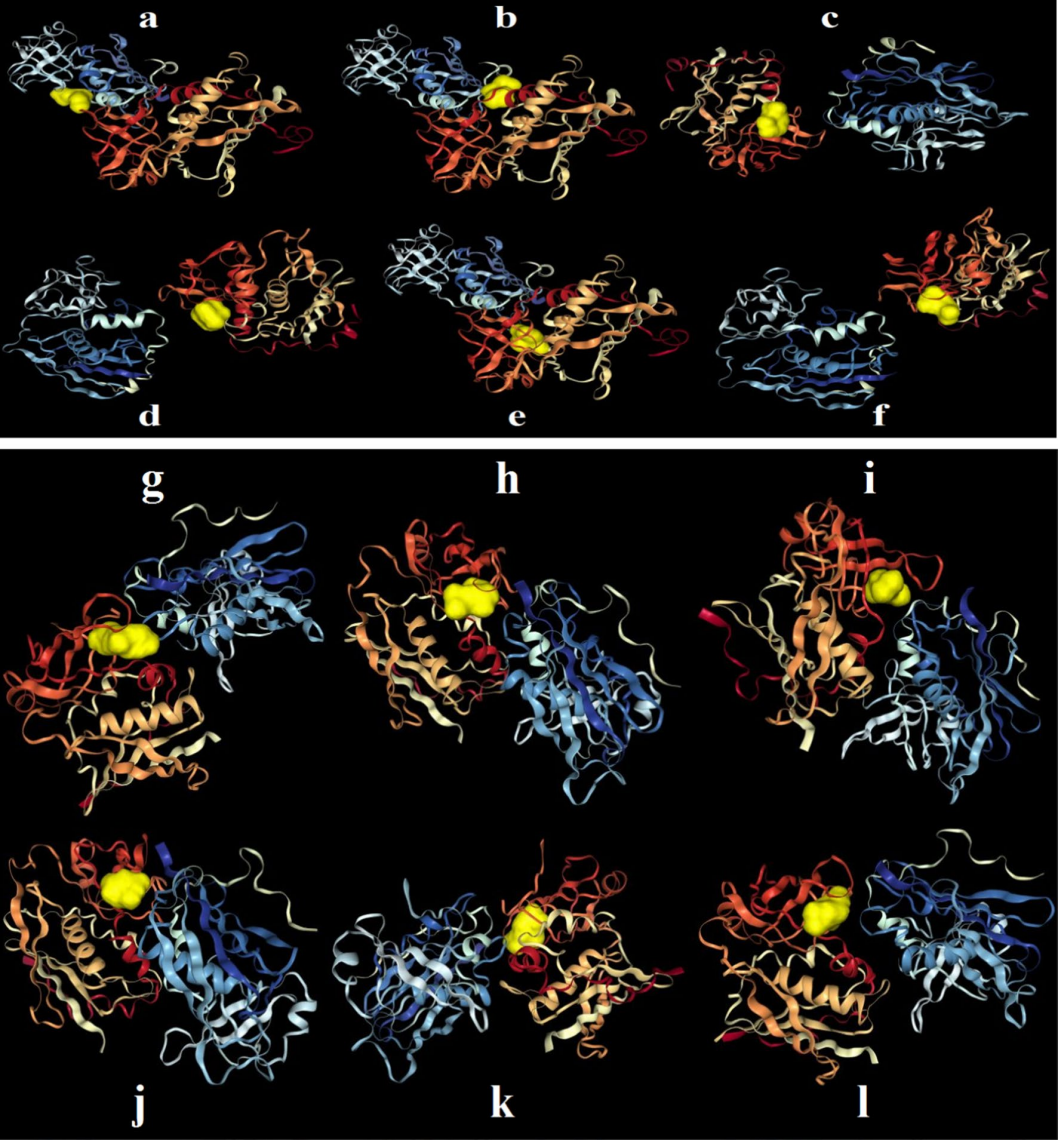
Docking of (*Z*)-α-Bisabolene epoxide (**a**), (*E*)-Nerolidol (**b**), α-Cyperone (**c**), daphnauranol C (**d**), nootkatin (**e**), and nootkatone (**f**) with enterotoxin A. Docking of (*Z*)-α-Bisabolene epoxide (**g**), (*E*)-Nerolidol (**h**), α-Cyperone (**i**), daphnauranol C (**j**), nootkatin (**k**), and nootkatone (**l**) in yellow color with enterotoxin B

Table 3 presents type of amino acids interacting with each enterotoxin (A and B) by their binding affinity values. In the respect to enterotoxin A, highest and lowest affinity were respectively observed for daphnauranol C and (E)-Nerolidol by − 6.2 and − 4 kcal/mol. However, the difference of affinity between other compounds was not significant. Similarly, HDOCK confirmed this result as more negative docking score of − 107.46 for daphnauranol C with RMSD of 23.77 Å. In the case of daphnauranol C, HIS50, LEU48, PHE47, ASP70, ARG214, TYR108, LEU68, ALA97, TYR92, and GLN95 interacted with enterotoxin A. Docking poses illustrated at Fig. 5, wherein tyrosine, asparagine, and histidine were common interacted amino acids with enterotoxin A. Best affinity was found for nootkatin with value of − 6.7 kcal/mol towards enterotoxin B, which (E)-Nerolidol showed lowest one by − 4.6 kcal/mol. Amino acids of PRO1006, LYS1007, PRO1008, ASP1009, GLU1010, LEU1011, THR1184, TYR1186, TYR1233, and THR1235 were responded to docking of ligand of nootkatin with receptor of enterotoxin B at hydrogen bond and steric interaction (Table 3 and Fig. 5). In addition, HDOCK showed similar affinity for nootkatin with docking score of − 111.67 and ligand RSMD of 51.17 Å (Table 4). According to HDOCK result, docking interactions of each compound additionally are presented in Figs. 6. Betulin and 28-Norolean-12-en-3-one as triterpene metabolites showed free energy values of − 147.39 and − 83.97 kcal/mol toward staphylococcal enterotoxin A, respectively [62]. In another study, for the natural metabolite of catechin (flavan-3-ol), there was binding affinity between the hydroxyl group at position 3 of the galloyl group and the active sites staphylococcal enterotoxin A [63]. Autodock scores were − 48.4, − 41.4, − 42.2, and − 35.5 kJ/mol for (-)-epigallocatechin-3-gallate, (-)-epigallocatechin, kaempferol-3-glucoside, and kaempferol, respectively [64]. Additionally, as antibacterial mechanism, these terpenoides can hinder growth of bacteria by the disruption of the cellular membrane integrity [65].

**Table 3:** Interacting amino acids and binding affinities (Kcal/mol) for six natural metabolites toward enterotoxin toxin A and B

Ligands	Enterotoxin A	Interacting residues for enterotoxin A	Enterotoxin B	Interacting residues for enterotoxin B
(*Z*)-α-Bisabolene epoxide	− 5.2	GLN19, GLY20, TYR64, GLY93, TYR 94, GLN95, CYS96, ASN102, and THR104	− 5.6	PHE1044, LEU1045, ARG1065, GLU1067, TYR1089, GLN1092, TYR1094, SER1096, LYS1097, and SER1211
(*E*)-Nerolidol	− 4	LYS10, ILE7, GLU2, LEU183, SER1, ASN128, ASP227, VAL185, ASN195, SER193, HIS187, and HIS225	− 4.6	TYR2091, CYS2093, TYR2094, PHE2095, SER2096, ASP2108, and LYS2111
α-Cyperone	− 5.3	ASN33, GLY93, TYR94, ASN25, SER206, and TYR205	− 6.6	PRO1006, LYS1007, PRO1008, ASP1009, GLU1010, LEU1011, THR1184, TYR1186, TYR1233, and THR1235
Daphnauranol C	− 6.2	HIS50, LEU48, PHE47, ASP70, ARG214, TYR108, LEU68, ALA97, TYR92, and GLN95	− 6.4	LYS1025, ASP1029, VAL1169, LYS1170, LYS1173, TYR1175, GLU1176, LYS2170,
Nootkatin	− 5	HIS50, ARG214, ARG211, TYR108, ASP70, LEU48, PHE47, ASN207, ALA97, and TYR92	− 6.7	PRO1006, LYS1007, PRO1008, ASP1009, GLU1010, LEU1011, THR1184, TYR1186, TYR1233, and THR1235
Nootkatone	− 5.6	THR38, LYS37, PHE57, THR59, PHE58, ALA36, LYS35, TYR91	− 5.9	PHE2044, LEU2045, ARG2065, GLU2067, TYR2094, SER2096, and LYS2097

**Table 4:** Results of HDOCK indicating docking score and ligand RMSD in the best mode of docking

Ligands	Enterotoxin A		Enterotoxin B	
	Docking score	Ligand RMSD (Å)	Docking score	Ligand RMSD (Å)
(*Z*)-α-Bisabolene epoxide	− 88.30	51.24	− 101.46	51.31
(*E*)-Nerolidol	− 91.86	27.62	− 92.54	50.99
α-Cyperone	− 88.98	26.36	− 99.64	51.03
Daphnauranol C	− 107.46	23.77	− 101.88	49.72
Nootkatin	− 99.03	26.45	− 111.67	51.17
Nootkatone	− 89.49	25.57	− 98.01	51.16

### Interaction of albumin and hemoglobin with CuNCs

**Fig. 7:**
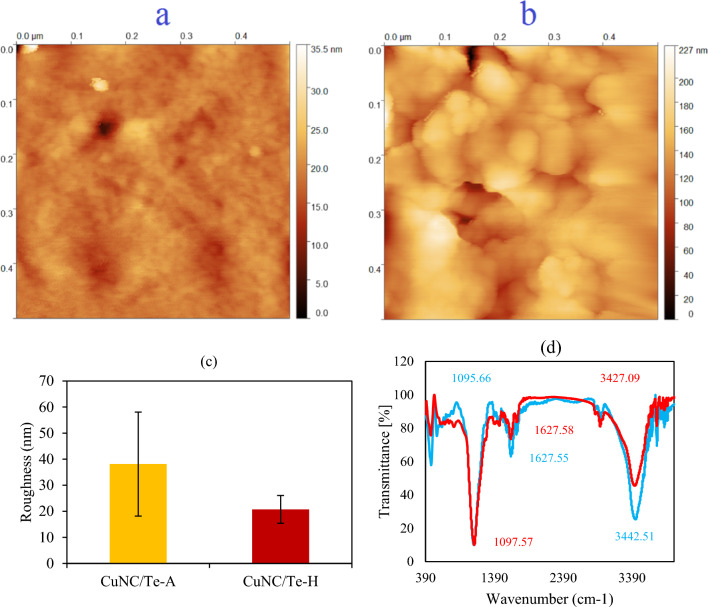
AFM results of CuNC/Te-A (**a**) and CuNCs/Te-H (**b**) with related roughness (nm) ± SD for each nanocomplex (**c**), and **d** FTIR spectra for CuNC/Te-A (blue line) and CuNC/Te-H (red line)

**Fig. 8:**
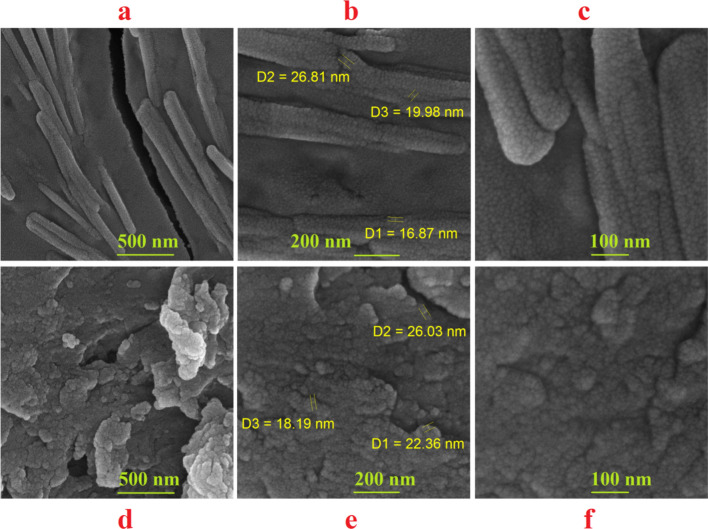
SEM photographs for the interaction of albumin (**a**–**c**) and hemoglobin proteins (**d**–**f**) with CuNPs-Te NCs

Interaction of green prepared CuNCs with two main blood proteins including albumin and hemoglobin was evaluated by FTIR, AFM, and SEM techniques. Size and concentration of proteins can impact on roughness and self-assembly of proteins around the metal NPs or NCs [66], wherein larger size of protein may result in a higher aggregation and roughness. In this regard, size of human serum albumin and hemoglobin are 8 − 13 nm and 5–6.9 nm, respectively [67, 68], which CuNC/Te-A showed roughness of 38.12 ± 19.96 nm compared to CuNC/Te-H having 20.74 ± 5.33 nm (Figs. 7a–c). Electrophoretic mobility of these proteins can be changed by binding of metal ions to the metal binding sites, which can induce a protein aggregation. As reported in a previous study, a total molar ratio of 8 metal ions of cobalt to one human serum albumin can promoted aggregation of these proteins [40]. As presented in Fig. 8, SEM images showed rod shape of CuNC/Te-A (diameter = 109.26 ± 40.30 nm and length = 1055.64 ± 271.62 nm) in contrast to globular shape of CuNC/Te-H by average size of 321.75 ± 194.28 nm. Spectra of FTIR exhibited main peaks at 3442, 2919, and 1095 cm^−1^ for functional groups of –O–H, –C–H and –C–O stretch, separately (Fig. 7d). There was higher intensity of transmittance for CuNC-Te-A relative to CuNC-Te-H showing a major role of –O–H group in creating of complex of CuNC-Te-A.

## Conclusion

In according to SEM micrographs, increased permeability, deformation, and destruction of bacterial envelope for both Gram-positive and Gram-negative were observed under treatment of CuNCs/Te as main antibacterial effect. CuNCs containing biogenic carbon/FeSO_4_/Cu/CuO can form pores and facilitate uptake of tetracycline by bacteria and therefore synergize antibiotic effect. In this way, antibacterial activity of CuNCs/Te can be the most probable pathway relative to each CuNCs and tetracycline agent alone. Entirely, synergistic activity of bio-fabricated CuNCs with antibiotics may be affected by main parameters of amount and ratio of antibacterial agents, physicochemical properties of CuNCs, bacterial type (Gram-negative or Gram-positive), antibacterial functions, and chemical structure of antibiotics. More analysis by molecular docking of these functionalized NCs approved a good binding affinity in the case of daphnauranol C and nootkatin towards virulence factors of enterotoxin A and B, respectively. In association with self-assembly behavior of these NCs, higher roughness of CuNC-Te-A was indicated compared to CuNC-Te-H by showing a major role of –O–H group in producing of complex of CuNC-Te-A. This property is a critical affair to form core–shell formulation of these NCs with main blood plasma or interaction of these NCs in physiological conditions, which in these cases; more studies particularly in vivo are needed.

## Research highlights

- Antibacterial activity of CuNCs/Tetracycline can be more efficient relative to CuNCs and tetracycline agent alone.
- Synergistic activity of bio-fabricated CuNCs with antibiotics may be affected by main physicochemical properties.
- There was a good binding affinity in the case of daphnauranol C and nootkatin towards virulence factors of enterotoxin A and B, respectively.
- Higher roughness of CuNC-Tetracycline-Albumin was indicated compared to CuNC-Tetracycline-Hemoglobin.

## Data Availability

Data will be provided upon request.
